# Defect-Engineered Heat Transport in Graphene: A Route to High Efficient Thermal Rectification

**DOI:** 10.1038/srep11962

**Published:** 2015-07-01

**Authors:** Weiwei Zhao, Yanlei Wang, Zhangting Wu, Wenhui Wang, Kedong Bi, Zheng Liang, Juekuan Yang, Yunfei Chen, Zhiping Xu, Zhenhua Ni

**Affiliations:** 1 Jiangsu Key Laboratory for Design and Fabrication of Micro-Nano Biomedical Instruments, School of mechanical engineering, Southeast University, Nanjing 211189, China; 2 Department of Physics and Key Laboratory of MEMS of the Ministry of Education, Southeast University, Nanjing 211189, China; 3 Applied Mechanics Laboratory, Department of Engineering Mechanics and Center for Nano and Micro Mechanics, Tsinghua university, Beijing 100084, China; 4 Graphene Research and Characterization Centre, Taizhou Sunano New Energy Co., Ltd. Taizhou 225300,China

## Abstract

Low-dimensional materials such as graphene provide an ideal platform to probe the correlation between thermal transport and lattice defects, which could be engineered at the molecular level. In this work, we perform molecular dynamics simulations and non-contact optothermal Raman measurements to study this correlation. We find that oxygen plasma treatment could reduce the thermal conductivity of graphene significantly even at extremely low defect concentration (∼83% reduction for ∼0.1% defects), which could be attributed mainly to the creation of carbonyl pair defects. Other types of defects such as hydroxyl, epoxy groups and nano-holes demonstrate much weaker effects on the reduction where the *sp*^2^ nature of graphene is better preserved. With the capability of selectively functionalizing graphene, we propose an asymmetric junction between graphene and defective graphene with a high thermal rectification ratio of ∼46%, as demonstrated by our molecular dynamics simulation results. Our findings provide fundamental insights into the physics of thermal transport in defective graphene, and two-dimensional materials in general, which could help on the future design of functional applications such as optothermal and electrothermal devices.

Graphene features a superior phononic thermal conductivity *κ* = ∼5300 W/mK, which is attributed to both its high group velocities of acoustic phonons, *v* = 23.6, 15.9 m/s for LA, TA branches, and long phonon mean free path *l*_MFP_ = 240 nm^1^,^2^,^3^,^4^. Moreover, the two-dimensional monatomic structure of graphene allows every carbon atom to be engineered, which suggests an ideal test-bed for the study of thermal transport in solids by considering the disorder and imperfection. In the previous work, it has been reported that defects such as vacancies, isotopic doping, and chemically functional groups at low concentration act as localized phonon scatters, which reduce the thermal conductivity^5^,^6^,^7^. Experimental studies show that ∼1% ^13^C isotope atoms reduce *κ* by ∼25%^7^. Theoretical and computational studies suggest that vacancy-like and *sp*^3^-type defects can reduce the thermal conductivity up to tens of times at a certain areal coverage^8^,^9^,^10^. As the concentration further increases, the material then becomes more like an amorphous solid, or phonon glass, with a reduced mean free path for the heat carrier on the order of a few interatomic spacings^11^, and the limit of minimum of thermal conductivity is reached. In this situation, heat is conducted via hopping between localized atomic vibrations^12^,^13^,^14^. Graphene oxide with oxygen-rich functional groups, which can be tuned upon chemical treatments, pushes for this amorphous limit of thermal conduction at high defect concentration^4^,^10^,^15^,^16^. However, the knowledge about thermal transport between the crystal and amorphous limits is still very limited. To bridge the gap between graphene and its amorphous counterparts, it is crucial to understand the mechanism of heat transport in defective graphene (DG), *e.g.* how is the reduction in conductivity related to the type and concentration of defects? How could the defect engineering of heat transport be implemented for functional devices with the capability of modulating heat flow? Answering these questions could further enable relevant technical applications.

In this work, we study the thermal conduction in graphene with the presence of a broad spectrum of defects by performing molecular dynamics (MD) simulations. We find that carbonyl pairs and vacancies lead to more significant reduction in the conductivity than hydroxyl, epoxy groups and nano-holes. This conclusion is confirmed by our non-contact opto-thermal Raman measurements, where defects are introduced through oxygen plasma irradiation and a huge reduction of *κ* = ∼83% is characterized at an extremely low defect concentration of ∼0.1%. We then explore the possibility to manipulate 2D heat conduction and flow by patterning defects into grapehene by performing MD simulations, and demonstrate a remarkable thermal rectification ratio of ∼46% through a junction between pristine and DG with carbonyl pairs.

## Results

### The effectiveness of defects in reducing the thermal conductivity of graphene

**Figure 1:**
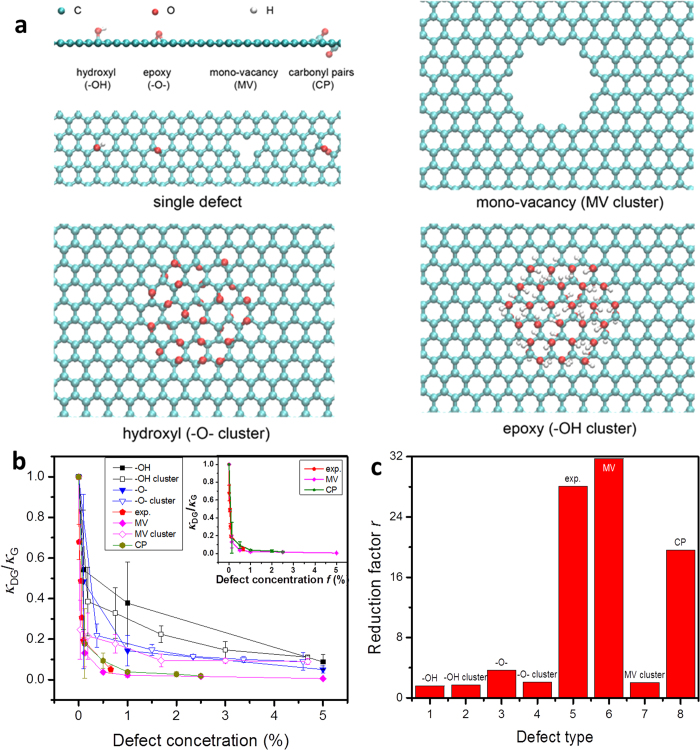
(**a**) Typical defect types characterized in DG that include hydroxyl, epoxy groups, carbonyl pairs and mono-vacancies. Isolated defects of these types and their clusters (except for the carbonyl pair clusters that are unstable) are both considered in the simulations. (**b**) Thermal conductivities of DG (*κ*_DG_) measured in the unit of *κ*_G_ for the pristine graphene sheet with various types of defects. The ones with mono-vacancies and carbonyl pairs are singled out in the inset, along with the experiment results. (**c**) The reduction factor *r* calculated for various types of defects with the concentration of 0.1% measured in the simulations and experiments following the effective medium theory.

Defects could be created in graphene by exotic treatments such as oxygen plasma irradiation or chemical functionalization. Typical defects that have been characterized in oxidized graphene include hydroxyl, epoxy groups, carbonyl pairs, vacancies, as well as nanoholes^17^,^18^,^19^,^20^,^21^. Here, to explore their effects in modifying thermal transport behaviors in graphene, we firstly adopt MD simulations to calculate the in-plane thermal conductivities of graphene containing four different types of defects (Fig. 1a), which will be compared with our opto-thermal Raman measurements. The thermal conductivities are calculated using the Green-Kubo formula, which relate the thermal conductivity of materials with the auto-correlation function of heat flux operators (see Methods for details). In general, our simulation results in Fig. 1b demonstrate that these defects can be categorized into two sub-groups as proposed in the Lerf-Klinowski model for graphene oxide^22^. The hydroxyl and epoxy groups do not break the underlying hexagonal lattice and preserve relatively well the lattice symmetry of carbon atoms and integrity, thus disturb the thermal transport weakly. In contrast, the presence of carbonyl pairs (CP) and vacancies reduce the thermal conductivity of graphene significantly as they break the in-plane network of *sp*^2^ carbon bonds.

To quantify the concentration dependence, we fit our MD simulation results empirically by using the effective medium theory (EMT)^9^, where the thermal conductivity of DG *κ*_DG_ is related to the thermal conductivity of defects *κ*_d_, pristine graphene *κ*_G_, and the areal defect concentration *f* as:


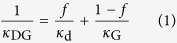


By rewriting Eq. 1 into *κ*_DG_/*κ*_G_ = 1/(1 + *rf*), we can define a reduction factor *r* = *κ*_G_/*κ*_d_ −1 that quantifies the strength of reduction in thermal conductivity, or equivalently an effective size of the defect *a* = *l*_G_ − *l*_d_ = *rl*_G_/(*r* + 1), where *l*_G_ and *l*_d_ are the mean free paths by considering the phonon-phonon scattering processes in pristine graphene and defect scattering in DG, respectively. The results summarized in Fig. 1c indicate that strong perturbation from the carbonyl pairs and mono-vacancies reduce *κ*_DG_ significantly (with a one-order higher values of *r*), in stark contrast to the much weaker effects of hydroxyl and epoxy groups that are uniformly distributed in the samples. We also find that once the hydroxyl and epoxy groups clustered into groups, the reduction in *κ* is almost unchanged. In contrast, the scattering of mono-vacancies is significantly weakened by their coalescence into nano-holes, where scattering occurs at the edges of nanoholes instead of discrete point defects. These results provide an argument that the defects breaking in-plane *sp*^2^ bonds are more effective in reducing the thermal conductivity of graphene.

Thermal conductivity in a solid can in general be expressed in the form of kinetic theory, which is expressed as:


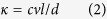


where *c* is the specific heat, *v*, *l* = *vτ*, *τ* are the average phonon group velocity, mean free path, relaxation time, and *d* is the dimension of problem. The scattering sources arising from defects in graphene include mass difference, local strain and boundaries. If we assume that the relaxation times for all these scattering processes are independent, the phonon relaxation or scattering time *τ* can be calculated by the Matthiessen’s rule *τ*^−1^ = Σ_*i*_*τ*_*i*_^−1^, where the relaxation time *τ*_*i*_ characterize all scattering mechanisms including the phonon-phonon scattering and defect scattering^23^. The previous work suggests that the absorption of oxygen groups does not significantly change the group velocities, and thus the thermal conductivity is mainly reduced by the shortening of mean free path in the regime of defect scattering^4^. However, defects that break the in-plane *sp*^2^ lattice integrity not only introduce stronger scattering centers for propagating phonons, but also reduce the group velocities by softening the lattice^9^. This contrast explains why carbonyl pairs and vacancies lead to higher reduction of the thermal conductivity in graphene.

### Thermal conductivity of oxygen-plasma-treated graphene

**Figure 2:**
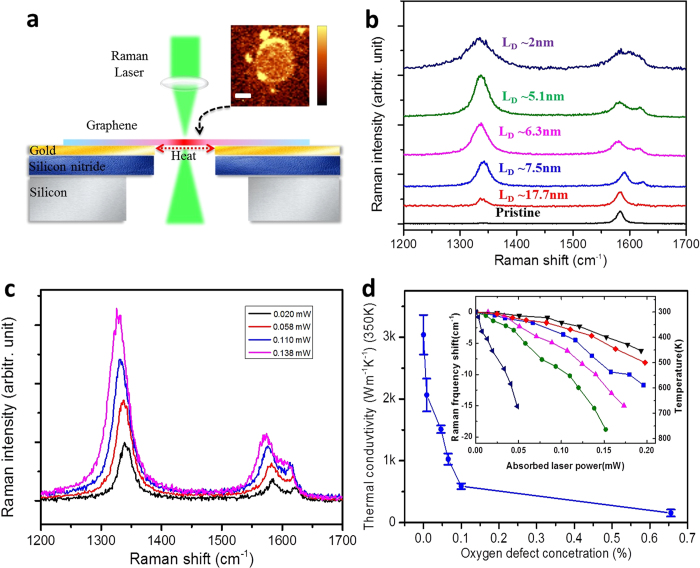
(**a**) Schematic of the experiment, where graphene film is covered on the 50 nm-gold-coated silicon nitride membrane with a 3.0 μm diameter hole. Inset is Raman mapping of the intensities of G peak, scale bar is 1 μm. (**b**) Raman spectra of monolayer defective graphene after oxygen plasma irradiation. The *L*_*D*_ stands for inter-defect distance in graphene. (**c**) Raman spectra of oxygen plasma treated graphene under different absorbed laser power. (**d**) Thermal conductivities of oxygen plasma treated graphene with different defect concentration. The inset is the G peak frequency shift as a function of the absorbed laser power.

To verify our simulation results, the thermal conductivity of oxygen-plasma-treated graphene was measured by using a non-contact optothermal Raman technique. Oxygen plasma irradiation creates oxygen-containing functional groups and a certain amount of vacancies in graphene^24^. The basic setup of thermal conductivity measurement is illustrated in Fig. 2a^1^,^25^. The Raman spectrum of pristine CVD monolayer graphene^26^ presents an intense peak at ∼1580 cm^−1^, namely the G peak^27^. Raman mapping integrated from G peak intensity confirms the graphene film across the 3.0 μm hole is homogenous and continuous without any visible wrinkles or broken holes, as shown in the inset of Fig. 2a. Plasma-treated graphene possesses additional D and D’ peaks (Fig. 2b), which originate from the double resonant Raman process with the presence of defects^28^. The intensity ratio between the D and G peaks, *I*_*D*_*/I*_*G*_, is commonly used to estimate the defect concentration^29^. It can be clearly seen that the I_D_/I_G_ increases with the plasma power density (Stage 1), and decreases after a certain point (Stage 2). Here, Stage 1 indicates the structural transition from high quality graphene to defective graphene and Stage 2 refers to the transition from defective graphene to amorphous carbon^30^. To estimate the density of defects, the inter-defect distance *L*_*D*_ follows the relation of Eq. 3 in Stage 1^29^,^30^,^31^:


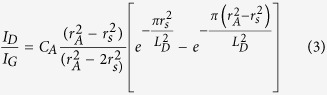


where *C*_A_ ∼4.2 is a parameter given by previous experiment results^29^. *r*_S_ ∼1 nm determines the radius of the structurally disordered area, and *r*_A_ ∼3.1 nm is defined as the radius of the area where the D peak scattering takes place^29^. In our case, the *I*_D_/*I*_G_ of oxygen plasma treated sample is about ∼0.4, ∼1.8, ∼2.3, ∼3.0 and ∼2.2 (Stage 2) when the absorbed laser power is 0.1 mW, corresponding to an average defect distance of ∼17.7, ∼7.5, ∼6.3, ∼5.1 and ∼2 nm (based on Ref. 31), respectively. Accordingly, the concentration of oxygen-containing defects is ∼0.1, ∼0.4, ∼0.6,∼1.0, and ∼6.6‰.

In our opto-thermal Raman experiments, the increase in local temperature at the laser spot causes a redshift of Raman peaks (Fig. 2c)^32^,^33^, because of the thermal expansion and 3- and 4-phonon scattering processes^27^. Therefore, local temperature at the measured spot can be plotted as the function of absorbed laser power of graphene, and adopted to extract the thermal conductivity *κ*^25^. Typical results for G frequency shift as a function of the absorbed laser power for samples with different oxygen defect concentration are plotted in the inset of Fig. 2d. Larger frequency shift indicates a higher temperature at the laser spot and hence a lower thermal conductivity of the sample. It can be clearly seen that the temperature of samples with higher defect concentrations rises much faster compared to those with lower defect concentrations under the same laser power. According to the thermal transport model in Cai *et al.*’s work^25^, the temperature distribution in suspended graphene *T*(*r*) is:


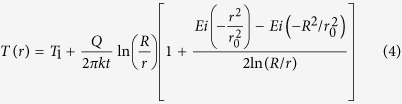


where *T*_1_ is the temperature at the boundary of suspended graphene, *i.e.* the part in contact with the gold film, which is assumed to keep at room temperature. *Q* is the absorbed laser power in total and *R* = 1.5 μm is the radius of suspended graphene. *t* = ∼0.34 nm is thickness of graphene, and *r* is radial position from the laser beam’s center. *Ei*(*x*) is exponential integral and *r*_0_ = 0.15 μm is the radius of measured laser beam. In this work, the thermal coefficient for G peak of 4.05 ± 0.2 × 10^−2^ cm^−1^/K is adopted for the thermal conductivity data fitting^25^, and we find that the thermal coefficients of DG are similar to that of pristine graphene and almost independent on the defect concentration. As shown in Fig. 2d, the pristine graphene has a thermal conductivity of 3.50 ± 0.32 × 10^3^ W/mK at 350 K, which is comparable to previously reported values^1^,^7^,^25^. On the other hand, the thermal conductivities of oxygen-plasma-treated graphene drop to 2.06 ± 0.27, 1.48 ± 0.06, 0.93 ± 0.09, 0.59 ± 0.05 and 0.16 ± 0.06 × 10^3^ W/mK with the increase of defect concentration. The calculated reduction factor *r* from experimental results is comparable to the values predicted for lattice vacancy and carbonyl pair by our MD simulations, as shown in Fig. 1b,c. It is then suggested that the above two types of defects (the vacancy and carbonyl pair) could be the main contributors to the great reduction of thermal conductivity in oxygen plasma treated graphene.

It is interesting to find that in experiments, with the defect concentration of 0.1%, the thermal conductivity of graphene has dropped by ∼83%. While as suggested by our MD simulation results, such a reduction requires for a much higher defect concentration when the defect type being hydroxyl, epoxy groups, as well as nano-holes^34^. This reduction is also more significant compared to those observed in experiments by considering the effect of substrates^35^, isotope doping^7^, organic residues^36^, and metal nanoparticles attachment^37^, which reduce *κ* much more gently. It should be mentioned that the measured thermal conductivity of graphene could be differed by experimental details, such as the effects of absorptance on graphene, the temperature coefficient of G peak^1^,^7^,^25^,^38^, and techniques of measurement^36^,^37^. However, the reduction factor *r* should be a reliable measurement since all graphene samples are measured under same condition and the influence of absorptance and temperature coefficient is minimized via the calculation of *κ*_DG_/*κ*_G_.

### Self-repairing of vacancy-like defects

**Figure 3:**
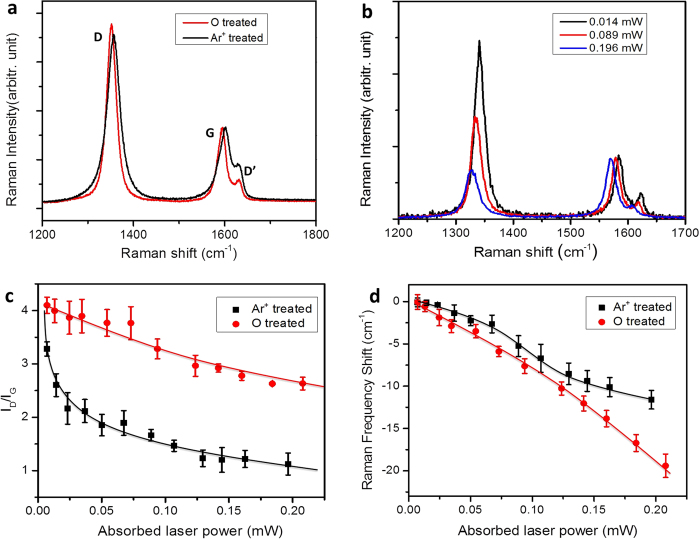
(**a**) Raman spectra of Ar^ + ^ and oxygen plasma treated graphene. (**b**) Raman spectra of Ar^ + ^ plasma treated sample under different absorbed laser power. (**c**) I_D_/I_G_ of Ar^ + ^ and oxygen plasma treated graphene as a function of absorbed laser power. (**d**) The G peak frequency shift of Ar^ + ^ and oxygen plasma treated graphene as a function of the absorbed laser power.

In order to verify whether the vacancy-like defects play an important role in thermal conductivity reduction in our experiment, we study the sample treated by Ar^ + ^ plasma, which is believed to introduce vacancy-like defects in graphene only. Previous work has demonstrated that the intensity ratio between the Raman D and D’ peaks (*I*_D_/*I*_D’_) can be used to distinguish the nature of defects in graphene, *e.g. I*_D_/*I*_D’_ is much higher for *sp*^3^-like defects (∼13) than vacancy-like defects (∼7)^39^. Figure 3a shows Raman spectra of Ar^ + ^- and oxygen-plasma-treated graphene, and *I*_D_/*I*_D’_ for these two samples (∼7.3 and ∼13.0) agree quite well with the above signature. With the increase of absorbed laser power, the *I*_D_/*I*_G_ for Ar^+^-plasma-treated-graphene dramatically decreases, as shown in Fig. 3b, indicating the decrease in defect concentration. In comparison, we plot the I_D_/I_G_ of Ar^+^- and oxygen-plasma-treated graphene as a function of the absorbed laser power in Fig. 3c. When the absorbed laser power increases to ∼0.2 mW, the *I*_D_/*I*_G_ of oxygen-plasma-treated-graphene dropped by ∼25% (from ∼4 to ∼3) only, while that of Ar^+^-plasma-treated-graphene dropped by ∼75% (from ∼4 to ∼1).

The above phenomena could be related to the self-repairing of vacancy-like defects. Previous work has shown that the vacancy-like defects tend to self-heal while being annealed in vacuum, which is attributed to the mechanisms of mobile carbon adatoms recombination with vacancies^40^, and the reconstruction of graphene lattice by forming non-hexagonal rings as topological defects^41^. For example, the mono-vacancy could reconstruct to form a five-membered and a nine-membered ring, while a di-vacancy could dissociate into two pentagons and one octagon. The self-repairing of vacancy-like defects could more complicated for laser heating in air (as in our experiments) compared to vacuum annealing^40^,^41^, *e.g.* nanoholes could be formed. Further work is required to elucidate the detailed atomic structures and reconstruction regimes.

The frequency shifts of Ar^ + ^- and oxygen-plasma-treated graphene with the increase in the absorbed laser power are plotted in Fig. 3d. According to the simulation results in Fig. 1c, the vacancy in lattice is an effective phonon scatterer^23^, and the frequency shift of Ar^ + ^-plasma-treated graphene should be larger than the oxygen-plasma-treated one. However, Fig. 3d suggest the opposite result – the average thermal conductivity of Ar^ + ^-plasma-treated graphene is ∼0.77 × 10^3^ W/mK, that is higher than the value of oxygen-plasma-treated graphene (∼0.55 × 10^3^ W/mK). Based on the above analysis, vacancy-like defects might not be the major source for the huge thermal conductivity reduction in our oxygen-plasma-treated graphene because of its strong self-repairing effect at high temperature. We suggest that oxygen groups leading to significant modification of the in-plane carbon-bonding network, such as the carbonyl pairs, are the main source of thermal conductivity reduction.

### Thermal rectifier using patterned graphene

**Figure 4:**
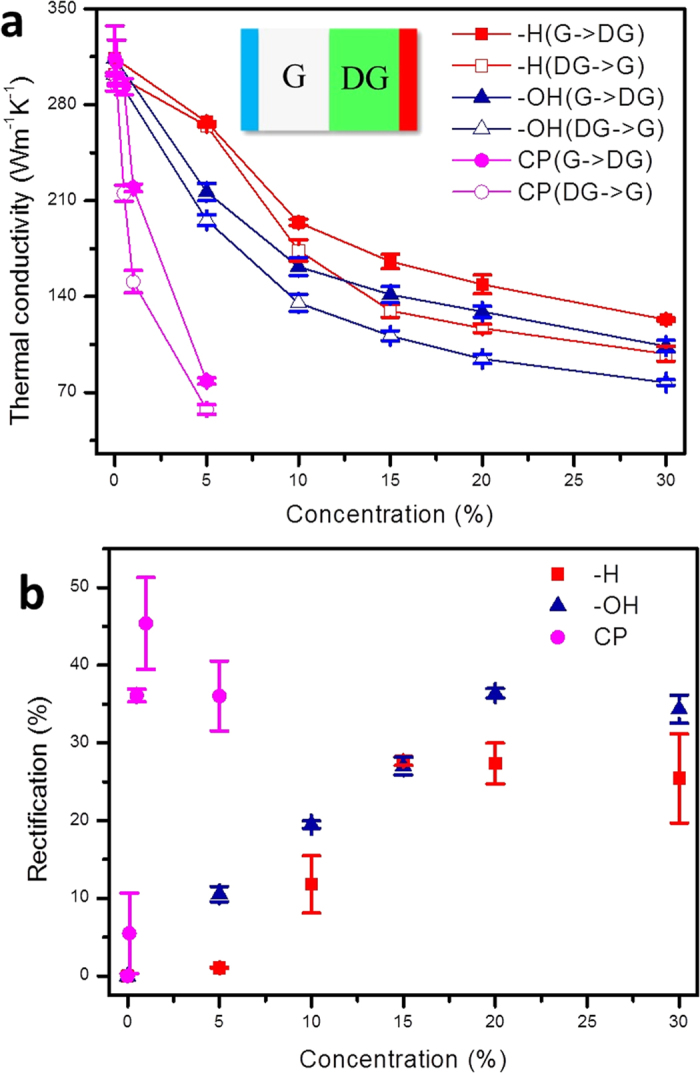
(**a, b**) Thermal rectification in a graphene-defective graphene junctions, where graphene is selectively functionalized by hydrogen, hydroxyl and carbonyl pairs with various concentration. The asymmetric in graphene and functionalized graphene induce difference in the thermal conductivity when the temperature gradient applied to the junctions is reversed (**a**) which yields a concentration-dependent rectification factor up to 46% (**b**).

As we have discussed, thermal transport in graphene can be controllably tuned by defect engineering, which offers a promising route to design functional devices with the capability of modulating the heat flow, *e.g.* the design of thermal rectification. Thermal rectifier is a two terminal device with a thermal conductance depending on the direction of heat flow, which has broad applications for heat control/management in the future^42^. Most experimentally reported thermal rectifiers require asymmetric shape^43^, mass density mismatch^41^ or interface between dissimilar materials^44^, which result in the sophisticated fabrication process and strongly limit the system stability. This aporia might be conquered by using functionalized or defective graphene^45^. Here, we propose an asymmetric junction by selectively introducing different types of defects or functional groups on one side and forming a graphene-defective graphene junction (G-DG junction). Our MD simulations show that for oxygen plasma treated graphene with carbonyl pair defects, the thermal rectification factor defined by *R*_th_ = (*κ*_G-DG_ – *κ*_DG-G_)/κ_DG-G_ could reach 46% at defect concentration *f* = 1% (Fig. 4), where *κ*_G-DG_ and *κ*_DG-G_ are the effective thermal conductivities when heat flux is directed from G to DG, and the opposite. This value of *R*_th_ converges with *f* after the peak is reached, and is significantly higher than the measured ratio of ∼7% obtained by coating boron-nitride nanotubes asymmetrically with metal particles, and the predicted ratio of ∼10% for asymmetrical graphene nanoribbons from MD simulations^42^,^46^. Similarly, the G-DG junction with other types of defects also demonstrate high rectification factors that is ∼27% for hydrogenated graphene and ∼36% for hydroxyl group functionalized graphene. However, the peak values are reached at a much higher defect concentration, due to the fact that their effects in modifying the thermal conduction in graphene are much weaker, which makes them less effective in practical applications compared to the carbonyl pair defects. From the results in Fig. 4b, we can see that the rectification ratio of CP features a significant drop at the defect concentration of 5%, which is different from other types of defects. This discrepancy may be attribute to the following fact. As the defect concentration approaches 5%, the CP defects that break C-C bonds and perturb the graphene lattice strongly could lead to lattice instability when they are located closely or overlap, which leads to the observed drop of rectification ratio. Previous works on G-DG junction as thermal rectifiers also consider vacancies, silicon substitutions, Stone-Wales defects^47^ or hydrogenation^48^ as manipulating means, while these types of defects are either not thermally stable or difficult to create. The origin of strong rectifying process of G-DG junction could be attributed to the fact that the vibrational density of states in graphene and DG is asymmetric^43^ and the low-frequency modes are more dominating in defective graphene due to the presence of defects and decrease in the stiffness^4^,^9^,^49^. As a result, the high-frequency vibrational modes carrying heat in pristine can propagate into DG more efficiently than their excitation by the energy flow from modes with lower frequencies, and thus the thermal conductivity from pristine graphene to the DG is higher than the reversed direction. It should be noted that even though the rectification factor is very high, this G-DG junction may still not be an ideal thermal rectifier, as heat flux in the low heat flux direction is not zero. Anyhow, this work sheds light on the single component system, which avoids the issue of interface thermal resistance and sophisticated fabrication process, and may open up its important applications in thermal management.

## Discussion

We have demonstrated using atomistic simulations and non-contact optothermal Raman technique that the carbonyl pairs in oxygen-plasma-treated graphene could lead to a huge reduction of thermal conductivity of graphene, with ∼83% at defect coverage of only ∼0.1% compared to pristine one. Such a great reduction could not be explained by commonly considered defects in oxidized graphene with oxygen-rich groups such as the hydroxyl and epoxy groups. Moreover, we have also shown that, vacancy like defects, although predicted to greatly reduce the thermal conductivity, might not be the major contributor either because the strong self-repairing effect. The significant reduction of the thermal conductivity could be attributed mainly to the creation of carbonyl pair defects. With the ability to control the selective functionalization of graphene (e.g. introducing carbonyl pairs by oxygen plasma), we propose a thermal rectifier with high rectification ratio ∼46%, at only 1% defect concentration. Our results could also help on the better understanding of mechanism of thermal conductivity reduction in graphene-based materials, and also provide an efficient way to tune the local thermal conductivity for the future design of graphene based devices, e.g. optothermal, electrothermal devices.

## Methods

### Experimental Section

#### Graphene synthesis and treatment

Monolayer graphene was synthesized on a copper foil (25 μm) by means of chemical vapor deposition (CVD) in a vacuum chamber, with a gas mixture of methane and hydrogen (60 and 90 sccm, respectively, total pressure of ∼300 Pa) at 1045 °C for 10 min^26^. A 3 × 3 array of 3.0 μm diameter holes was pre-fabricated in the 50 nm-gold-coated silicon nitride membrane using focused ion beam. The graphene film was transferred onto the membrane to form suspended structure. Defective graphene samples were fabricated via oxygen or Ar^ + ^ plasma treatment (commercial 13.56 MHz RF source). The defect concentration can be modulated by fixing the plasma power (15 W, 5 s for oxygen plasma and 40 W, 60 s for Ar^ + ^ plasma) and changing the gas pressure (from 5 Pa to 40 Pa, to change the plasma density).

#### Opto-thermal Raman measurement

Raman spectra were taken by a Witec alpha 300 R confocal Raman system with an excitation laser of 532 nm (2.33 eV). A 100x objective lens with numerical aperture of 0.9 is used, and the spot size of the laser is estimated to be ∼300 nm by scanning across a smooth cleaved edge of Au^25^. The Raman mapping of graphene is acquired with the step size of 100 nm and the result confirms that graphene are free from wrinkles or broken holes^1^,^25^,^50^. As informed from Ref. 1, the heat wave generated over the suspended portion of graphene on the 3.0 μm diameter hole continues to propagate all the way out. The variation in the heating power *Q* on the sample surface leads to the shift in the G peak position, which corresponds to change in the temperature at laser spot.

### Molecular Dynamics Simulations

#### Atomic structures

The molecular structure of DG consists of hydroxyl, epoxy, and carbonyl groups on the basal plane, as well as defective sites and open edges^22^. In this work, we construct models of DG containing hydroxyl, epoxy groups, as well as carbonyl pairs and mono-vacancies^17^. Their concentration *f* is defined as *n*_D_/*n*_C_ where *n*_*D*_ is number of the defective atom site and *n*_C_ is the number of carbon atoms in the pristine graphene lattice. The distribution of defects in DG is sampled randomly.

#### Molecular dynamics simulations

Classical MD simulations are performed using the large-scale atomic/molecular massively parallel simulator (LAMMPS)^51^. The all-atom optimized potential for liquid simulations (OPLS-AA) is used for DG, which can capture essential many-body terms in inter-atomic interactions, including bond stretching, bond angle bending, van der Waals and electrostatic interactions^52^. This force field was successfully applied in predicting thermal transport in graphene or carbon nanotubes^53^,^54^,^55^,^56^. Periodic boundary conditions are applied to a 9.82 × 8.52 nm^2^ supercell of graphene and DG, which are verified to capture the thermal conduction in G and DG structures^57^,^58^. The periodic box and atomic structures are relaxed in the simulations before investigating thermal transport.

#### Calculation of thermal conductivities

To compute the thermal conductivity *κ* of pristine and defective graphene from MD simulations, we use the Green-Kubo method based on the linear response theory^59^, which applies for systems in thermal equilibrium where heat flux fluctuates around zero. *κ* could thus be expressed as an integration of the heat flux operator multiplied by a prefactor


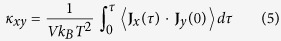


where *T* is the temperature of system, *k*_B_ is the Boltzmann constant, and *V* is the system volume that is defined here as the area of graphene or DG multiplied by a nominal thickness (the inter layer distance of graphite, 3.4 Å). The upper limit of time integration *τ*_c_ needs to be long enough so the current-current correlation function decays to zero, *J*_*x*_ and *J*_*y*_ are the heat current operators in the *x* and *y* directions, and the angular bracket represents the ensemble average, namely the heat flux autocorrelation function (HFACF). The heat flux **J** of the system is computed from the expression **J** = (Σ_*i*_**e**_*i*_**v**_*i*_ – Σ_*i*_**S**_*i*_**v**_*i*_)/*V*, where *e*_*i*_, **v**_*i*_ and **S**_*i*_ are the total energy, velocity vector, and stress tensor of each atom *i*, respectively. We first integrate HFACF with an integration time *τ* following Eq. 5, then obtained the relation between *κ* and *τ*. The decorrelation time for the heat flux is typically on the scale 100 ps for our models, and thus converged results for *κ* could be extracted when *τ > τ*_c_ in the simulations. The advantage of Green-Kubo method, compared to the non-equilibrium molecular dynamics (NEMD) simulations with thermal gradient built up across the material that is used in our investigation on the non-equilibrium processes, is that it does not need to perturb the simulated system out of equilibrium, where nonlinearity arises^60^. Moreover, the Green-Kubo approaches demonstrates a weaker size dependence^60^,^61^. Due to the hexagonal symmetry of graphene lattice and random distribution of defects, the thermal conductivity of is isotropic. We thus evaluate *κ* as the mean value of *κ*_*xx*_ and *κ*_*yy*_ that may differ in the finite system under simulation though, where the maximum difference between *κ*_*xx*_ and *κ*_*yy*_ in our simulations is less than 0.1*κ*.

In the MD simulations, the atomic structures of graphene or DG are firstly equilibrated at the same temperature as the experimental condition (*T* = 350 K, where the quantum nuclear effect is weak) under a Nosé-Hoover thermostat for 200 ps, in which the time step is 0.1 fs. The stress is released by coupling with a barostat. The structure is further equilibrated in a NVE ensemble for 50 ps before the thermal conductivity is calculated. In the equilibrium Green-Kubo calculations, the system is simulated in a microcanonical ensemble as well. The atomic positions and velocities are collected during the simulations to evaluate the heat flux and its autocorrelation functions. The thermal conductivity is finally obtained by following the Green-Kubo formula. The results are calculated by averaging over five different runs for the same DG sample at a certain defect concentration.

#### Simulations for the thermal rectification

In simulating the thermal rectification in a G-DG junction, we construct a graphene strip with length of 19.6 nm and width of 5.2 nm with in-plane periodic boundary conditions applied, and half of the strip along the length direction is functionalized by uniformly distributed hydrogen, hydroxyl groups or carbonyl pairs with various concentrations. The thermal conductivity is calculated by following Müller-Plathe’s NEMD approach in a NVE ensemble^62^. The strip is partitioned axially into slabs for temperature recording and control, after heat flux *J* is induced by exchanging the momentum between the ‘hottest’ atom in sink slab and the ‘coldest’ atom in the source slab. The effective thermal conductivity in both directions of the junction is calculated by following the Fourier’s law^63^


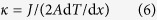


The cross-sectional area *A* is calculated by considering the thickness of graphene and DG as 3.4 Å. We assume that the temperature gradient d*T*/d*x* is Δ*T*/*d*, where Δ*T* and *d* are the temperature difference and distance between heat source and heat sink, respectively.

## Additional Information

**How to cite this article**: Zhao, W. *et al.* Defect-Engineered Heat Transport in Graphene: A Route to High Efficient Thermal Rectification. *Sci. Rep.* **5**, 11962; doi: 10.1038/srep11962 (2015).
